# Improved Multichannel Electromyograph Using Off-the-Shelf Components for Education and Research

**DOI:** 10.3390/s22103616

**Published:** 2022-05-10

**Authors:** Enrico M. Staderini, Stefano Mugnaini, Harish Kambampati, Andrea Magrini, Sandro Gentili

**Affiliations:** 1 Healthy World Association, 1400 Yverdon-les-Bains, Switzerland; 2 Occupational Medicine Section, Department of Biomedicine and Prevention, “Tor Vergata” University of Rome, 00133 Roma, Italy; s.mugnaini@med.uniroma2.it (S.M.); andrea.magrini@uniroma2.it (A.M.); sandro.gentili@uniroma2.it (S.G.); 3 Doctorate School in Industrial Engineering, “Tor Vergata” University of Rome, 00133 Roma, Italy; harish.kambampati@students.uniroma2.eu

**Keywords:** EMG, electromyography, Arduino, signal acquisition, signal conditioning

## Abstract

Most students and researchers with limited funding are often looking for simple and low-cost devices for the acquisition of the electromyogram signal (EMG) in an educational or research setting. Thus, off-the-shelf devices are used and they have already been described in the literature, but they are used without considering their real performances, which are, in general, quite poor from the electronic and signal processing points of view. It is the purpose of this communication to present the evidence of these issues, and to describe an improved version of the “classical” duo, composed of the common ECG/EMG Olimex board and the Arduino microprocessor board. In this case, the Arduino-DUE is used. Three main points are highlighted in this paper: (a) the bandpass characteristics of the ECG/EMG Olimex board and how they can be improved to cope with EMG bandwidth requirements; (b) the increase in sampling frequency of the signal; and, finally, (c) the possibility of automatic detection of more ECG/EMG Olimex boards installed at the same time as the shields on the Arduino-DUE board. Very simple and low-cost modifications on the ECG/EMG Olimex board could deliver a much better performing multichannel EMG acquisition system, suitable for educational classroom experiments and early proof-of-concept research.

## 1. Introduction

Most students and researchers are often in need of a simple electromyograph (EMG) system for the acquisition of muscle electrical signals for research or educational purposes. The acquisition of surface EMG signals is very well described in [1,2]. Due to limited funding, young researchers are always looking for very simple and cheap devices. Arduino computers are simple and cheap enough, and easily available for the purpose, along with the Olimex ECG/EMG Arduino shields [3], so they appear to be quite good candidates to create, easily and rapidly, a working research EMG system. Unfortunately, with the simple programming of Arduino, as described for naïve users, the acquisition of reliable signals is very difficult to obtain, unless proper programming is performed to speed up the sampling frequency. Furthermore, the straightforward use of the Olimex ECG/EMG shield, although cheap, is technically not adequate for the acquisition of EMG raw signals, as will be demonstrated in this work. Indeed, the bandpass of the original Olimex board, which is limited to 100 Hz as adequate for the ECG signal, is not sufficient for EMG signal acquisition, which requires a much larger bandwidth. The International Society for Electrophysiology and Kinesiology (ISEK) standard [4] defines the frequency content of the surface EMG signal as between 5 Hz and 450 Hz, which means that the signal amplifier’s acquisition chain for the EMG should guarantee this bandwidth. It is the purpose of this study to provide instructions to modify the Olimex board, so that it minimally complies with ISEK’s standards for surface EMG signal acquisition. Further descriptions of surface EMG signal quality assurance are given in Gerdle et al. [5].

Indeed, the coupling of an Arduino board (Arduino UNO, Arduino DUE, Arduino Mega, Ivrea, Italy) with an Olimex ECG/EMG board (Olimex Ltd. Plovdiv, Bulgaria) has permitted many researchers to develop a large series of devices to be used for educational or research projects. Shah et al. [6] used an Arduino-Uno board with a self-made bioelectrical amplifier, but they established a bandpass of only 245 Hz for the EMG signal, which is quite a low bandwidth for a signal with quite a high frequency content. Ullah Khan et al. [7] describe a MyoWare device whose bandwidth is not defined, not even on its own data sheet, as it is mostly used to obtain a rectified and filtered EMG signal for gaming purposes. Rojas et al. [8] used the Olimex/Arduino duo for gait analysis, but no need for a large bandwidth was declared, as the signal was band-limited, rectified and filtered after acquisition. Rahman et al. [9] described the development of biosignal acquisition shields for Arduino architectures, but they also band-limited the signal to 150 Hz. More correctly, Logesh Kumar et al. [10] used a custom-made biosignal amplifier, which, according to the schematics given in their paper, should have adequate bandwidth characteristics. Duc Minh Dao et al. [11], along with Tariquzzaman et al. [12], used a filtered-rectified EMG signal, as did other authors [13,14].

Clearly, the use of a plug-and-play EMG shield, such as the Olimex board, is very practical, and this is the solution often followed by many students and young researchers. We also considered the use of this architecture, but, after due analysis of the electrical characteristics of the commonly available ECG/EMG boards to be used in connection with Arduino boards, and the performances of Arduino sketch programming for data acquisition, we considered that there is still something to gain by obtaining a higher-quality EMG recording system with enough signal fidelity, not only for the classical EMG rectified-filtered analysis, but also for raw EMG signal analysis, while maintaining a low-cost instrument suitable for simple educational and research purposes.

Thus, it is the scope of the present communication to describe a hands-on, slightly modified combination of the Arduino–Olimex system for EMG, which will be able to provide reliable EMG signals for research or didactic purposes by young researchers and students.

Suitable modifications of ECG/EMG Olimex shields are described in detail, and software for EMG acquisition with a high sampling rate is also provided. A very simple, cheap and useful, but high-quality, EMG signal acquisition system is now available to anybody, following the instructions given in this communication.

## 2. Description of Board Modifications and Associated Programming

Four ECG/EMG Olimex shields mounted on an Arduino DUE board [15] were used. The acquired signals were sent using a USB connection to a laptop computer for real-time presentation and recording.

There are two major problems in using ECG/EMG Olimex shields and Arduino DUE. The first is the very limited bandwidth of the Olimex shield, as it comes from the factory, which is not adequate for a minimally reliable EMG signal recording. Indeed, the original Olimex ECG/EMG board bandwidth was found to be limited to 100 Hz, which is adequate for ECG, but not for EMG. The second is the very low sampling frequency, which is attainable using the standard programming of Arduino DUE. The following paragraphs will describe which hardware modifications must be performed on the Olimex shields to improve their bandwidth, and how to program the Arduino DUE for faster sampling. The results will be shown using an in-house developed Windows-based software, which is not described nor provided in this work. As a matter of fact, most researchers just need to deliver the EMG signal from the Arduino to a MATLAB script on a computer.

### 2.1. How to Modify the Olimex ECG/EMG Shield

**Figure 1:**
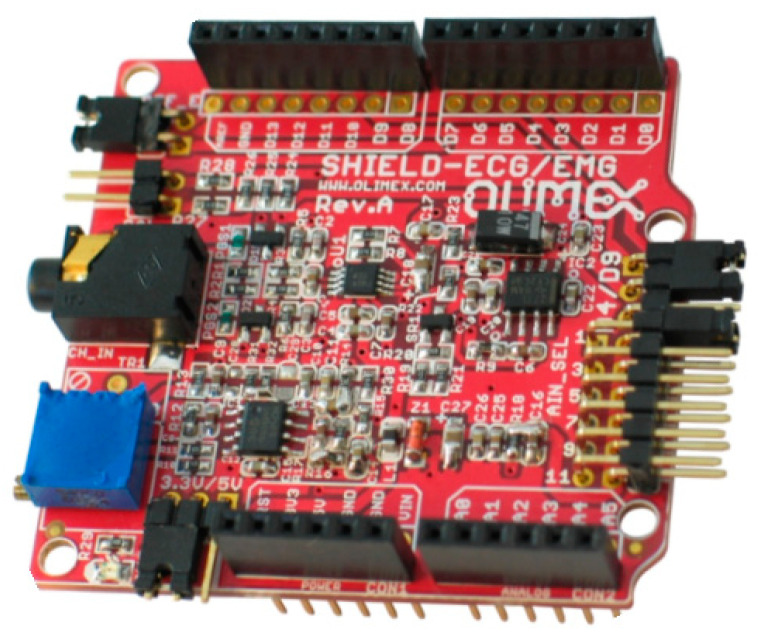
The Olimex ECG/EMG Arduino shield (image from Olimex ECG/EMG shield manual).

The Olimex ECG/EMG shield shown in Figure 1, as provided by the factory, has a bandwidth that is too narrow to obtain a minimally acceptable EMG signal. The original bandwidth of the Olimex ECG/EMG board can be shown by simulating the original circuit using a SPICE (“Simulation Program with Integrated Circuit Emphasis”) program. SIMetrix from SIMetrix Technologies (https://www.simetrix.co.uk/ accessed on 22 October 2021) was used, whose free version is downloadable from their website, and, for this reason, it was very often used by the students.

**Figure 2:**
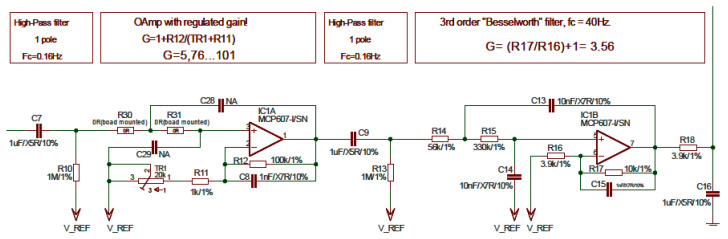
Schematics of single-ended amplifier (after the instrument amplifier) on the Olimex ECG/EMG shield (image from Olimex ECG/EMG shield manual) before modification.

**Figure 3:**
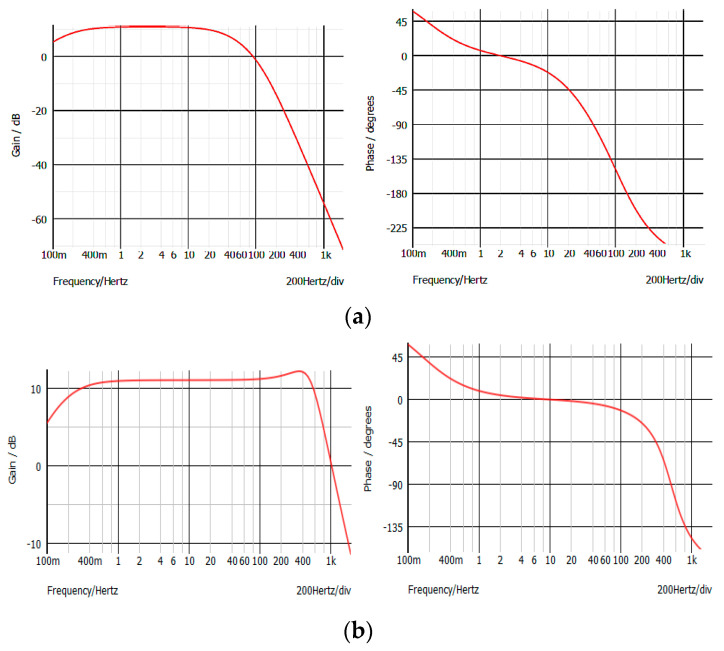
(**a**) Bode plot of original Olimex ECG/EMG shield amplifier (as computed with SIMetrix). (**b**) Bode plot of MODIFIED Olimex ECG/EMG shield amplifier, where a much wider bandwidth is obtained, and this is suitable for EMG recording.

Figure 2 depicts the original ECG/EMG amplifier, which comes from the factory, as reported on the Olimex board. Capacitors C28 and C29 are not soldered on the original board; thus, the section using IC1A is not responsible for bandwidth limiting. On the contrary, the section using IC1B provides a low-pass 40 Hz corner frequency. The section with integrated circuit IC1B was simulated to ascertain the module and phase characteristics, as shown in Figure 3a.

The Bode plot simulation shown in Figure 3a confirms the characteristics given by the producer. The frequency content of a standard surface EMG is normally expected to be in the bandwidth of 5 to 500 Hz, with the main energy content being dominant between 50 and 150 Hz; thus, the Olimex ECG/EMG board bandwidth, while being adequate for ECG recording, is clearly insufficient for EMG signals.

**Figure 4:**
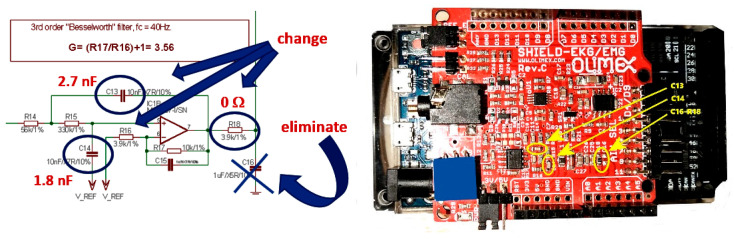
Changing the values of components for widening Olimex ECG/EMG shield amplifier bandwidth (note the typo on the original Olimex document: “Besselworth” instead of Butterworth).

A decision was made to modify the original Olimex board to change the values of C13 and C14, to eliminate C16, and, eventually, to change the value of R18. The components to change/modify/eliminate are indicated in Figure 4.

Warning! One must admit that desoldering and resoldering of the surface mount components on the Olimex board is not a very simple task, and a proper soldering iron and a surface microscope might be needed. With the modifications indicated, the new bandwidth of the amplifier is modified as shown in Figure 3b.

In another trial, the calibration circuitry available on the Olimex ECG/EMG shield was simulated and a square wave signal was found to be produced on the calibration pins of the board. With a 3.3 V PWM signal from the Arduino board (indicated as CAL_SIG on the original schematics), the obtained calibration signal has an AC peak–peak amplitude of 165 µV with a 7.5 µV DC component. These values agree with those stated by the factory in the manual.

**Figure 5:**
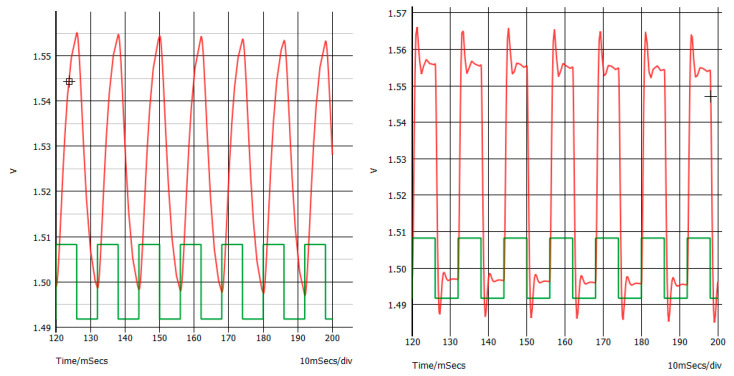
On both sides, the green tracing is the input to the simulated original or modified last stage amplifier of the Olimex board, and the red tracing is the output of the amplifier. Left side: original Olimex ECG/EMG amplifier output. Right side: modified amplifier showing faster slew rate and emphasis (overshot) of the rising signal, which is much more adapted to amplify EMG signal MUAPs or EMG interference signal. Note that overshot can be eliminated using a value of 1.8 nF for the C13 capacitor.

The time-domain calibration signal amplification by the original circuitry was simulated to be compared with the signal produced by the modified circuitry. To do so, it was supposed that a 100 times gain was being produced by the instrumentation amplifier, along with the subsequent amplifier stage driven by IC1A (the actual gain depends on the TR1 variable resistor). Thus, in the simulator, a 16.5 mV peak–peak square wave signal was applied to the input of the original, and to the input of the modified stage as well, to appreciate the effect of a low-pass bandwidth on the signal. The results are reported and described in Figure 5.

Another modification to the ECG/EMG shield is made by adding six 10 Kohm resistors in parallel to the six analog pins on the board.

The rationale for the use of these resistors is to impose pull-down, which may be used to automatically differentiate the analog input pins driven by an ECG/EMG shield from the empty (i.e., not driven) analog input pins. In this way, there will be no need to change the software on Arduino, regardless of how many and which ECG/EMG shields are used (provided that each shield will drive only one analog input pin).

Warning! This modification should be made on only one shield if you will use more than one ECG/EMG shield on the Arduino DUE, as this is performed to develop an automatic software detection of ECG/EMG shields installed on Arduino DUE.

**Figure 6:**
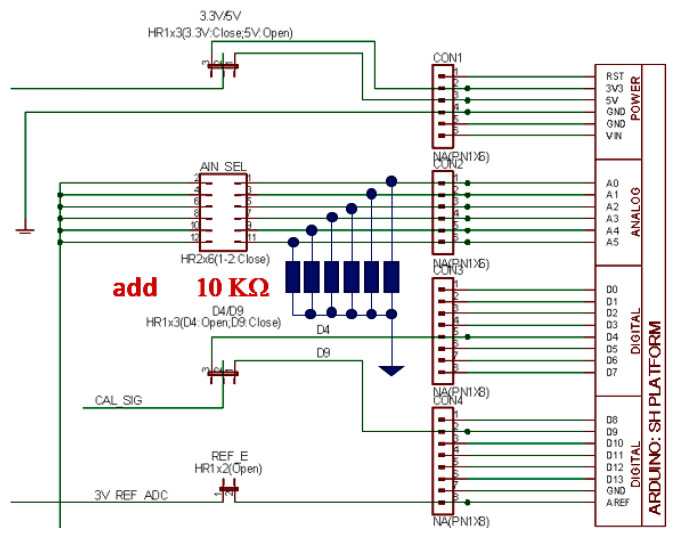
Application of pull-down resistors on the analog pins of the ECG/EMG shield.

Figure 6 shows the schematics where the pull-down resistors must be soldered onto the main board. The “main” board is the only Olimex ECG/EMG board with the same pull-down resistors. Indeed, only one board will use the pull-down resistors, which will work for the other boards as well, without lowering and, consequently, charging the output pin of the IC1B too much.

This way, the hardware modifications to the Olimex ECG/EMG shield are over. In the following section, the Arduino DUE board and programming tricks will be presented in detail.

### 2.2. How to Develop a Fast Sampling Rate Arduino DUE Software

The Arduino DUE is a microcontroller board based on the Atmel SAM3X8E ARM Cortex-M3 CPU [16]. The Arduino DUE is the first Arduino board based on a 32-bit ARM core microcontroller. As correctly stated on the Arduino website, with 54 digital input/output pins and 12 analog inputs, it is the perfect board for powerful larger-scale Arduino projects.

Due to the high-frequency content of the EMG signal, a very high sampling frequency is needed, with respect to the Nyquist limit, to avoid aliasing. Thus, the decision was made to use a 1000 Hz sampling frequency. It must be said that this value is, in general, not adequate for a correct EMG signal recording; nevertheless, one should remember the low-level, student and preliminary research-grade purpose of this project.

Even with the use of quite a low sampling frequency of 1000 samples/sec, the standard function provided by the Arduino sketch framework, the well-known analogread(), appeared to be not fast enough.

Indeed, while it is true that the analogread() function may sample at frequencies as high as 10 Khz, when multichannel EMG acquisition is required, multiplexing and other program delays are there to significantly hamper the possibility of reaching very fast sampling rates.

Thus, the Arduino DUE must be directly programmed using the internal ADC registers of the Atmel CPU. The lines of programming are really simple, as explained in the following.

First, it must be noted that the ADC in the Arduino DUE is connected in such a way that the Arduino analog input pins A0, A1, A2, A3, … correspond to the AVR CPU ADC channels ch7, ch6, ch5, ch4, …, respectively. The converter can be programmed to acquire a sequence of channels in free run. The instructions for configuring the acquisition are given in section 43 of the SAM3x datasheet [15], although they are not simple to understand for a naïve user.

First, the ADC analog reference and the ADC resolution can be set as usual, as follows:

analogReference(AR_DEFAULT); // reference is 3.3 V (only option for DUE)

analogReadResolution(12);  // set ADC resolution to 12 bits

Second, the ADC control registers of ADC CPU must be set as follows (please note that the ADC structure is, by default, defined when the DUE is selected in the Arduino sketch):

ADC->ADC_CHER = 0xFC;  // channel enable register: enable ADC channels 2 to 7

ADC->ADC_CR = 2;       // control register: start analog to digital converter

ADC->ADC_MR |= 0x80;    // mode register: free run (never wait for any trigger)

At this moment, analog to digital conversions can be acquired from the ADC machine in the CPU channel data register (CDR), after checking that each conversion is complete on the interrupt status register (ISR):

while((ADC->ADC_ISR & 0xFC) != 0xFC); // wait for conversion complete

adcvalue = ADC->ADC_CDR[7−i];       // obtain the analog value on the DUE pin

And that is all! Other ADC registers are not considered because their default values after reset are normally good for the subsequent analog acquisition. The complete programming of Arduino is left to the reader, who will create the software that is most suitable for their needs.

One last caveat should be kept in mind: as soon as the programmer uses a standard analogread() instruction, the programming of the ADC machine just described will be changed and complete reprogramming of the ADC will be needed.

### 2.3. How to Develop the Software for the Automatic Detection of Installed ECG/EMG Shields 

The automatic detection of the installed ECG/EMG shields can be performed thanks to the modification (to be made on only one of the used shields) consisting of placing 10 Kohm pull-down resistors on the analog inputs, as described before.

It can be verified that the analog read of an open (i.e., not driven by a shield) analog pin on the Arduino DUE is commonly around 1000 to 3000 ADC units. On the contrary, when the open analog pin is pulled down with the 10 Kohm resistor, the obtained analog value is zero or very low. Finally, when the pulled-down analog pin is driven by the amplifier on the shield, the analog value (with electrodes not connected) is between 1700 and 2000 ADC units, because of the continuous voltage level present. Keeping in mind the previous findings, a very simple software can be developed on Arduino DUE for automatically and preliminary detecting the presence and position of each ECG/EMG shield used and installed on top of the Arduino DUE board.

## 3. Results

The authors are sure that the tips described in the previous paragraph will suffice for experimenters and researchers to obtain a research-grade EMG system suitable for most applications.

In our case, to show the results of the system created, a custom software, created for a particular application, is used. Both this software (written in Visual BASIC 6 under the Windows operating system) and the Arduino DUE sketch are not available for the readers of this paper; they are only used here to show the functions obtained by the methods and procedures described in the previous chapters.

### Final Hardware Appearance

**Figure 7:**
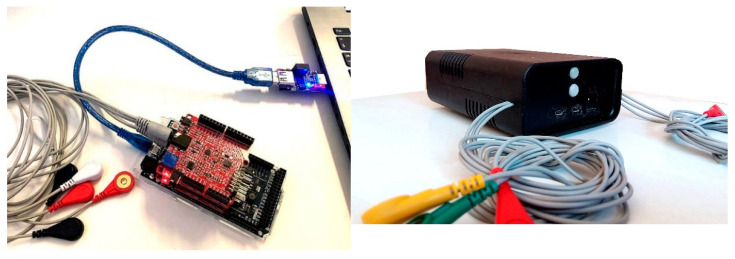
(**Left**) The Arduino DUE board with two modified Olimex ECG/EMG shields connected to the laptop USB socket using a 2.5 kV USB digital isolator based on Analog Devices ADuM3160 chip. (**Right**) The enclosed system ready to operate.

In Figure 7, the complete system is shown, composed of two Olimex Arduino shields on an Arduino DUE board. A USB isolator has been used for safety purposes between the laptop and the subject connected boards. This isolator provides complete galvanic isolation on the USB cable while transferring power from the computer to the Arduino/EMG system, and completing back and forth isolated communication of the USB serial data. The isolation device is currently on sale from major internet shops.

It must be clear that the resulting instrument is not certified for medical use, and it should only be considered as an RUO (research use only) device or educational device.

**Figure 8:**
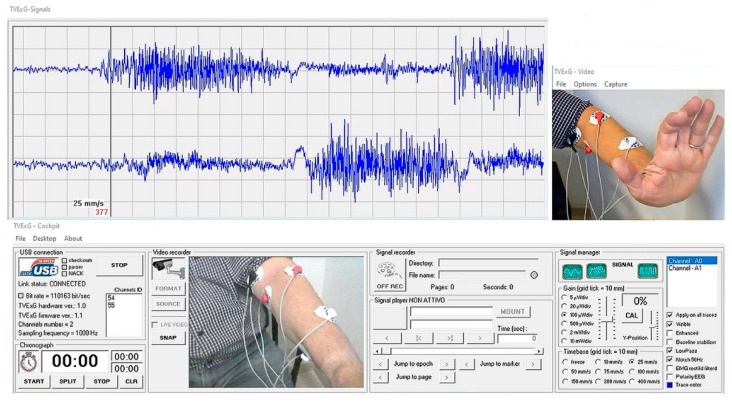
A screenshot of the signals coming from the built device.

A proprietary Windows software is used here (developed by the first author), and it is composed of quite an uncommon combination of Visual BASIC 6 and Windows 10 operating system. In Figure 8, the upper channel receives interference EMG signals from hand extension muscles, while the interference received by the lower channel is from hand flexion muscles at the forearm.

Images of the movement can be recorded synchronously with the tracings.

Actually, there are no clearly defined procedures to assess the surface EMG signal quality other than assuring that the acquisition system complies with certain technical requirements (such as ISEK’s standards).

## 4. Conclusions

While it should be stressed that this work is intended to be a technical communication, and not a complete research article, it must be underlined again that the device is for educational and research purposes, and it cannot be used for medical purposes. Nevertheless, this realization shows how very simple and cheap devices, such as the Arduino DUE and ECG/EMG Olimex boards, can be used effectively to obtain a working EMG device, which might be useful for student education and research. Many students and researchers were looking for such a simple device, but the results were not adequate, even for the simplest studies. The authors hope that this short communication will be very useful to them.
